# Glucocorticoids Cause Gender-Dependent Reversal of Hepatic Fibrosis in the MDR2-Knockout Mouse Model

**DOI:** 10.3390/ijms18112389

**Published:** 2017-11-10

**Authors:** Anca D. Petrescu, Stephanie Grant, Gabriel Frampton, Jessica Kain, Karam Hadidi, Elaina Williams, Matthew McMillin, Sharon DeMorrow

**Affiliations:** 1 Department of Medical Physiology, Texas A & M Health Science Center College of Medicine, Temple, TX 76504, USA; petrescu@medicine.tamhsc.edu (A.D.P.); sgrant@medicine.tamhsc.edu (S.G.); gframpton@medicine.tamhsc.edu (G.F.); jkain@medicine.tamhsc.edu (J.K.); khadidi@medicine.tamhsc.edu (K.H.); ecwilliams@medicine.tamhsc.edu (E.W.); 2 Central Texas Veterans Health Care System, Temple, TX 76504, USA; mcmillin@medicine.tamhsc.edu

**Keywords:** hypothalamic-pituitary-adrenal (HPA) axis, corticosterone, glucocorticoid receptor, hepatic cholestasis, liver fibrosis, cholangiocytes

## Abstract

Hepatic cholestasis is associated with a significant suppression of the hypothalamus-pituitary-adrenal axis (HPA). In the present study, we tested the hypothesis that activation of the HPA axis by corticosterone treatment can reverse liver inflammation and fibrosis in a multidrug resistance protein 2 knockout (MDR2KO) transgenic mouse model of hepatic cholestasis. Friend Virus B NIH-Jackson (FVBN) control and MDR2KO male and female mice were treated with vehicle or corticosterone for two weeks, then serum and liver analyses of hepatic cholestasis markers were performed. Indicators of inflammation, such as increased numbers of macrophages, were determined. MDR2KO mice had lower corticotropin releasing hormone and corticosterone levels than FVBN controls in the serum. There was a large accumulation of CD68 and F4/80 macrophages in MDR2KO mice livers, which indicated greater inflammation compared to FVBNs, an effect reversed by corticosterone treatment. Intrahepatic biliary duct mass, collagen deposition and alpha smooth muscle actin (αSMA) were found to be much higher in livers of MDR2KO mice than in controls; corticosterone treatment significantly decreased these fibrosis markers. When looking at the gender-specific response to corticosterone treatment, male MDR2KO mice tended to have a more pronounced reversal of liver fibrosis than females treated with corticosterone.

## 1. Introduction

The hypothalamus-pituitary-adrenal (HPA) axis consists of a complex set of signaling pathways at the neuroendocrine level, with roles in regulating physiological activities of various parts of the body for maintaining metabolic homeostasis in response to changes or stressors in the environment [1,2,3]. In clinical studies, an association of liver dysfunction, such as cirrhosis and obstructive jaundice, was found with adrenal insufficiency [4,5,6]. Furthermore, in experimental models of cholestasis, such as after bile duct ligation (BDL) in rats, the HPA axis was significantly dysregulated [7,8,9]. We have previously demonstrated that experimental suppression of the HPA axis in rats resulted in a similar increase in cholangiocyte proliferation as that observed after BDL and conversely, strategies to reactivate the HPA axis attenuated the biliary hyperplasia observed after BDL [9]. The suppression in the HPA axis during cholestasis has been partially attributed to the activation of the glucocorticoid receptor in the hypothalamus by aberrant bile acid signaling to suppress corticotropin releasing hormone [10].

An important step in HPA axis signaling is played by glucocorticoids and glucocorticoid receptors (GR), which mediate the signals from the adrenal glands to the tissues which are regulated by the HPA axis [11]. Interestingly, in animal studies, a gender-biased function of GR has been reported, e.g., in mouse liver GR differentially regulates inflammatory gene expression in males and females [12]. Another study in a sepsis model of systemic inflammation, specific genes had altered expression in response to glucocorticoids in both sexes but in opposite directions [13]. Gender-dependent differences of GR expression and function were demonstrated in the brain and liver of rats when exposed to stress [14,15]. Heat shock proteins (HSP) can bind to hepatic GR, thus preventing GR from binding DNA; in rats treated with antidepressants, HSP–GR binding was reduced in females but increased in males [14,15]. However, whether there is gender disparity in response to glucocorticoids in cholestatic liver diseases is unknown.

Based on our previous observations that the HPA axis, and especially hypothalamic function, was suppressed in rat models of hepatic cholestasis, we set out to determine (i) if the suppression of the HPA axis can also be found in other models of cholestatic liver disease; (ii) if treatment with glucocorticoids can attenuate liver cholestasis and subsequent biliary fibrosis; and (iii) if there is any gender disparity in the liver pathology or response to glucocorticoid therapy in a mouse model of cholestasis.

The MDR2KO mouse provides a model for studying hepatic cholestasis, liver fibrosis and hepatocarcinogenesis in the context of chronic inflammation [16]. The ablation of *AbcB4* gene encoding the MDR2 protein, a protein with function in phospholipid transport from hepatocytes into the biliary ducts, causes accumulation of toxic bile in the liver and subsequently hepatic cholestasis. Although the mechanism of pathogenesis in MDR2KO mice is not completely understood, several studies have demonstrated that mice lacking MDR2 protein develop a chronic inflammatory condition characterized by periductal inflammatory cell infiltration and periportal fibrosis [16,17,18]. In the present study, we treated male and female control (FVBN) and MDR2KO mice with vehicle (DMSO) or corticosterone. We tested markers of liver inflammation and fibrosis and found significant differences between males and females in the corticosterone-induced response.

## 2. Results

### 2.1. The HPA Axis Is Suppressed in MDR2KO Mice in Both Males and Females

**Figure 1:**
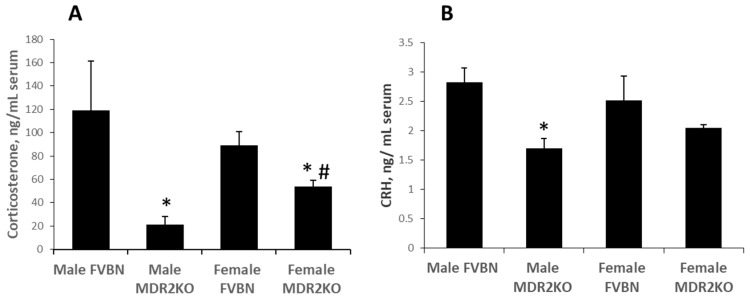
HPA axis markers in serum of male and female Friend Virus B NIH-Jackson (FVBN) control mice and MDR2KO mice. Corticosterone (**A**) and corticotropin-releasing hormone (CRH, (**B**)) concentrations were determined by enzyme immunoassay (EIA). The serum was collected from two-month-old male and female FVBN and MDR2KO mice. * MDR2KO versus FVBN. # females versus males; *p* < 0.05, *n* = 3.

To test the possibility that hepatic cholestasis in the MDR2KO mouse model is associated with changes in HPA axis function, we measured the level of corticosterone and corticotropin-releasing hormone (CRH) in serum of male and female control and cholestatic MDR2KO mice by enzyme immunoassay (EIA). As shown in Figure 1A, serum corticosterone concentrations were significantly lower in MDR2KO male and female mice as compared to FVBN control mice. Data also suggest that the decrease in corticosterone is more pronounced in male MDR2KO mice than in female. To determine if this suppression of glucocorticoid levels could be attributed to a dysregulation in hypothalamic signaling, circulating CRH levels were assessed. Concentrations of circulating CRH were significantly lower in MDR2KO males versus FVBN male mice, while in females there was the same trend even though it did not reach significance (Figure 1B). Thus, these results support a suppression of HPA axis in the MDR2KO mouse model of hepatic cholestasis with a higher degree of suppression in males than in females.

### 2.2. Liver GR Was Equally Expressed in Male and Female MDR2KO and FVBN Mice

**Figure 2:**
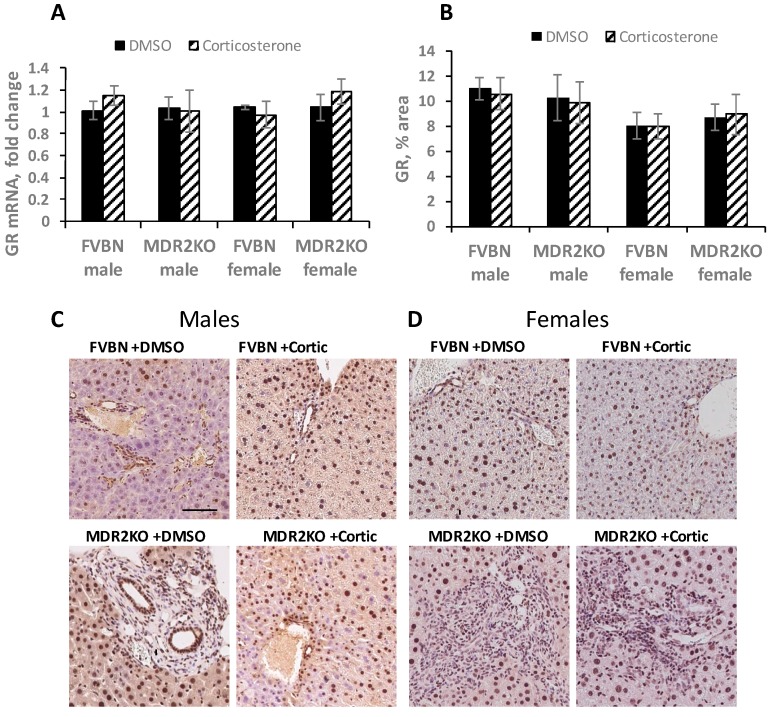
Glucocorticoid receptor (GR) expression in the liver of male and female FVBN and MDR2KO mice. (**A**) Results of RT-qPCR for GR performed with RNA isolated from livers of male and female FVBN and MDR2KO mice treated with vehicle or corticosterone; (**B**) The IHC images were analyzed and the percent areas of GR-stained pixels per field were estimated for each treatment group. *n* = 3, *p* < 0.05; (**C**,**D**) Representative IHC pictures of GR protein in liver sections from males and females FVBN and MDR2KO mice treated with vehicle or corticosterone. Scale Bar, 100 μm.

Based on the observation that the corticosterone levels were strongly reduced in MDR2KO mice as compared to controls, we hypothesized that treating MDR2KO mice with corticosterone may result in a reversal of HPA axis suppression and consequently a reversal of liver cholestasis. Since the main mediator of corticosterone signaling is GR, we assessed GR expression in the liver of MDR2KO mice and compared to controls treated with vehicle or corticosterone. Thus, GR expression was assessed by RT-qPCR and immunohistochemistry (IHC). GR mRNA was equally expressed in male and female FVBN and MDR2KO mice treated with either vehicle or corticosterone (Figure 2A). GR protein was abundantly expressed and concentrated in nuclei of hepatocytes and cholangiocytes in male and female FVBN control and MDR2KO mice (Figure 2B–D). These data suggest that GR is expressed in male and female MDR2KO mice at the same level as in FVBN counterparts.

### 2.3. Serum Chemistry of Male and Female MDR2KO Mice as Compared to FVBN Controls

**Figure 3:**
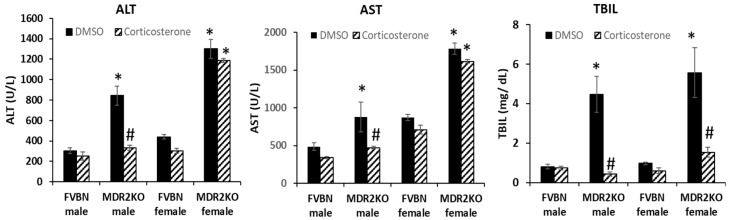
Serum chemistry of male and female FVBN and MDR2KO mice when treated with vehicle or corticosterone. Alanine aminotransferase (ALT), aspartate aminotransferase (AST) and total bilirubin (TBIL) were determined as described in Methods; *n* = 3, *p* < 0.05. * MDR2KO versus FVBN. # corticosterone versus DMSO.

Liver damage indicators such as aspartate aminotransferase (AST), alanine aminotransferase (ALT) and total bilirubin (TBIL) were assayed in the serum of male and female MDR2KO mice and FVBN controls treated with vehicle or corticosterone. The results are shown in Figure 3, and demonstrate that ALT and AST were increased in sera of MDR2KO mice of both genders as compared with FVBN controls. Corticosterone treatment reduced serum AST and ALT to normal values in male MDR2KO mice but not in females. Interestingly, bilirubin which was significantly higher than normal in MDR2KO mice males and females and its levels were reduced to FVBN values by corticosterone treatment in both male and female MDR2KO mice.

### 2.4. The Increased Intrahepatic Bile Duct Mass (IBDM) of MDR2KO Mice Was Reversed by Corticosterone Treatment

**Figure 4:**
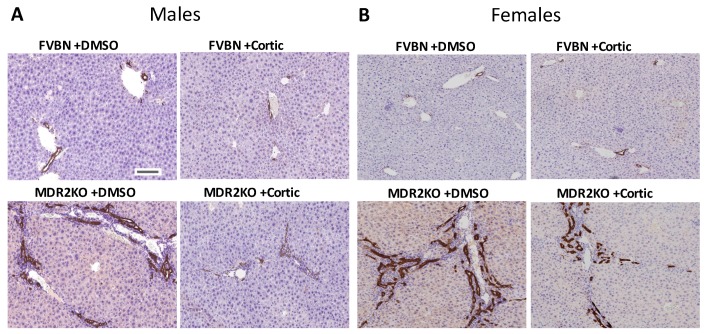

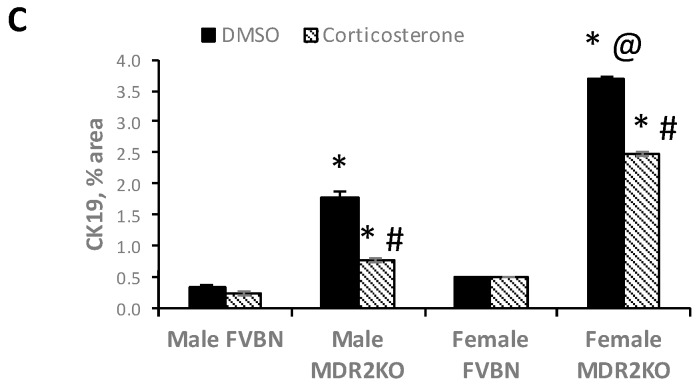
Intrahepatic bile duct mass (IBDM) is reduced by corticosterone in both male and female MDR2KO mice. Immunohistochemistry (IHC) images of cytokeratin 19 (CK19)-immunolabeled cholangiocytes in liver from male (**A**) and female (**B**) FVBN and MDR2KO mice treated with DMSO or corticosterone; (**C**) Percent area per field of CK19 staining in livers from male and female FVBN and MDR2KO mice treated with vehicle or corticosterone. * MDR2KO versus FVBN. # corticosterone versus DMSO treatment. @ females versus males; *n* = 3, *p* < 0.05. Scale bar, 100 μm.

There was a marked increase in cholangiocyte number per field, as demonstrated by cytokeratin 19 (CK19) immunoreactivity, in both MDR2KO male and female mice when compared to FVBNs although this effect was more pronounced in female mice (Figure 4). Furthermore, treatment with corticosterone resulted in an attenuation of the increased IBDM observed in MDR2KO mice with a stronger effect in males compared to females (a 56% reduction in males compared to a 20% reduction in females; Figure 4). These results indicate that corticosterone was able to reverse the massive accumulation of cholangiocytes in the MDR2KO mouse model of cholestasis, and this reversal is gender-dependent with corticosterone being more effective in males than in females.

In order to determine whether the inhibitory effect of corticosterone on IBDM in MDR2KO mice was due to increased cholangiocyte apoptosis or decreased proliferation of these cells, we assessed the apoptosis as well as the colocalization of proliferating cell nuclear antigen (PCNA), marker of cell proliferation, with the cholangiocyte marker CK19 in the liver of male and female FVBN and MDR2KO mice treated with vehicle or corticosterone.

**Figure 5:**
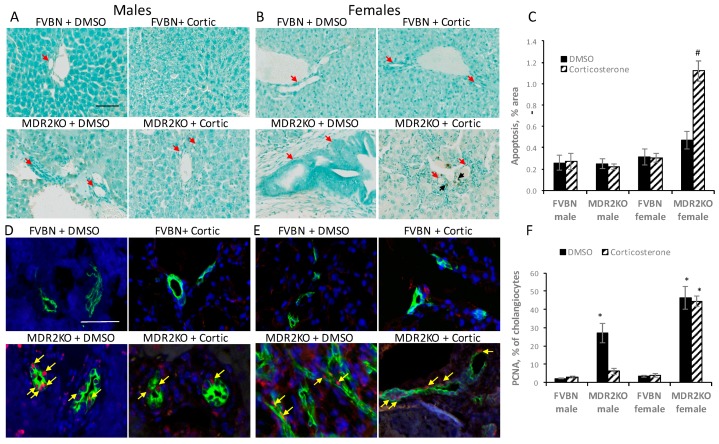
Effect of corticosterone treatment on apoptosis and cholangiocyte proliferation in the liver of MDR2KO mice. The extent of apoptosis in cholangiocytes was tested by TUNEL staining (terminal deoxynucleotidyl transferase dUTP nick-end labeling) in male (**A**) and female (**B**) FVBN and MDR2KO mice. Apoptotic cells appear in brown while the cells are counterstained with methyl blue. Cholangiocytes can be readily recognized by their characteristic shape (i.e., epithelial cells forming ducts, as indicated by red arrows). Black arrows demarcate apoptotic cholangiocytes. Colocalization of PCNA (proliferating cell nuclear antigen) and CK19 (marker of cholangiocytes) was tested by IF confocal microscopy of liver sections of male (**D**) and female (**E**) FVBN/MDR2KO mice when treated with vehicle or corticosterone. Representative images are shown in green being CK19 and in red, PCNA. The yellow arrows point to PCNA when present within cholangiocytes. Plots of apoptotic cholangiocytes as assessed through TUNEL images (**C**) and IF colocalization (**F**) of PCNA and CK19; *n* = 3, *p* < 0.05. * MDR2KO versus FVBN controls. # corticosterone versus DMSO. Scale bar, 100 μm.

Apoptotic activity was higher than normal in livers of female MDR2KO mice treated with corticosterone, but not in male MDR2KO mice undergoing the same corticosterone treatment (Figure 5A–C). The percentage of cholangiocytes (CK19-positive cells, Figure 5D–F) expressing PCNA was also determined in the liver of male and female MDR2KO and FVBN control mice. These data show that corticosterone reduced the number of proliferating cholangiocytes from 27% to 3% (normal level of FVBN mice) in male MDR2KO mice. In female MDR2KO mice, there was a higher percentage of proliferative cholangiocytes compared to male MDR2KO mice, and corticosterone treatment did not decrease cholangiocyte proliferation in female MDR2KO mice. Thus, it can be concluded that increased IBDM in female compared to male MDR2KO mice is due to a higher cholangiocyte proliferation rate in females. Furthermore, there is a gender-dependent mechanism for the reduction in IBDM observed after corticosterone treatment with an induction in apoptosis prevalent in females and the reduction in proliferation predominant in males.

### 2.5. Corticosterone Treatment Reduced the Excess Inflammatory Hepatic Cells in MDR2KO Mice

**Figure 6:**
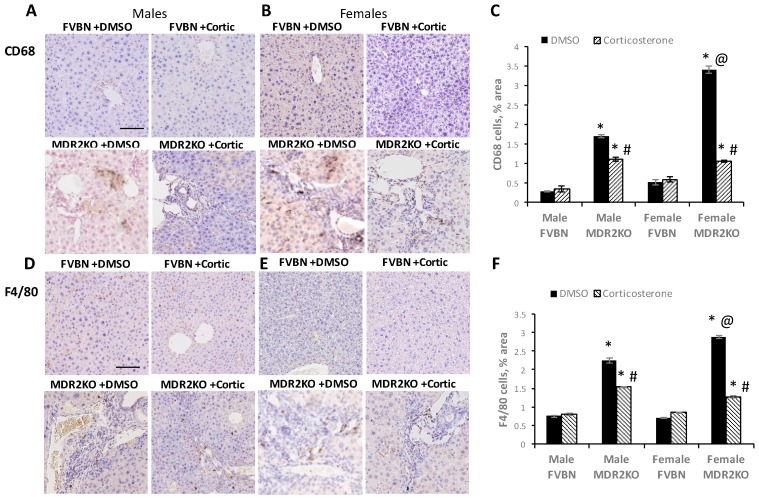
Corticosterone reduces liver inflammatory cells of male and female MDR2KO mice. IHC images of CD68^+^ cells in the liver of FVBN and MDR2KO mice, males and females, respectively (**A**,**B**). Plot of percent CD68^+^ cells per field quantified as percent area in the liver of male and female FVBN and MDR2KO mice (**C**). IHC images of F4/80^+^ cells in FVBN and MDR2KO mice, males and females, respectively (**D**,**E**). Plot of F4/80^+^ cells per field quantified in FVBN and MDR2KO mice, males and females (**F**). * MDR2KO versus FVBN mice. # corticosterone versus vehicle treatment. @ females versus males; *p* < 0.05, *n* = 3. Scale bar, 100 μm.

To further investigate the role of inflammation in the development of cholestasis in MDR2KO mice, we looked first at recruited monocytes/macrophages expressing CD68 and F4/80, which are known to be present in cholestatic liver [19,20,21] using IHC (Figure 6). It can be noted that there was a robust increase of CD68^+^ and F4/80^+^ macrophage numbers in male and female MDR2KO mice (Figure 6). In MDR2KO males, CD68^+^ cells were increased by six fold as compared to FVBN males and corticosterone treatment reduced the number of these inflammatory cells to 70% of MDR2KO mice treated with vehicle alone. In female MDR2KO mice there was a 6.5-fold increase in the number of CD68^+^ cells as compared to female FVBN mice, and treatment with corticosterone decreased this accumulation of CD68^+^ cells to approximately 30% of female MDR2KO mice treated with vehicle. Similarly, when measuring the changes in the number of monocytes/macrophages expressing F4/80 in liver of MDR2KO mice compared to FVBN mice, a three-fold increase in MDR2KO males was determined and this was reduced to 49% of vehicle with corticosterone treatment. In MDR2KO females, the hepatic F4/80^+^ cells were 4.1 fold more numerous than in FVBN females, and corticosterone treatment resulted in a 70% reduction of these cells as compared to vehicle-treated female MDR2KO mice (Figure 6).

**Figure 7:**
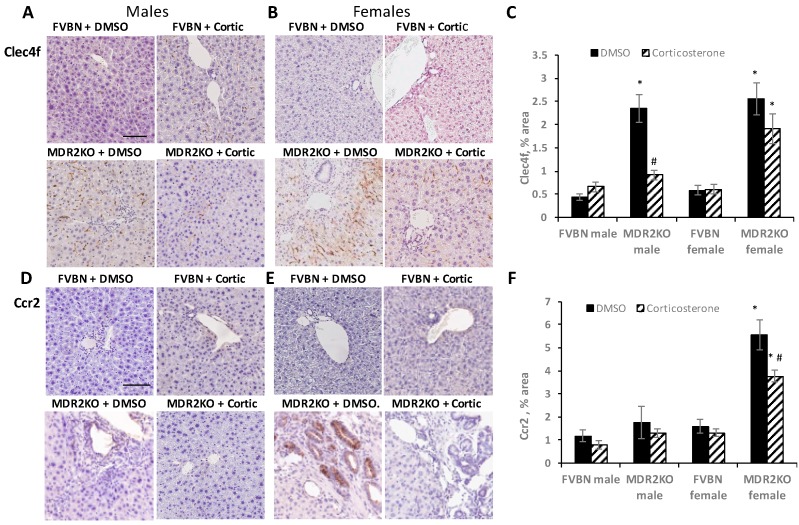
The effect of corticosterone treatment on Clec4f^+^ and Ccr2^+^ macrophages in liver of MDR2KO mice as compared to controls. Liver sections from male and female FVBN and MDR2KO mice which had been treated with vehicle or corticosterone were immunostained for Clec4f expressing cells (resident macrophages) and Ccr2 expressing cells (recruited macrophages). The IHC images were quantified and the results plotted. Pictures of Clec4f^+^ cells in liver of males (**A**) and females (**B**) FVBN and MDR2KO mice are shown. The plot of quantifications is in (**C**). Images of Ccr2-immunolabeled cells of male (**D**) and female (**E**) FVBN and MDR2KO mice were also quantified and the results are shown in (**F**); *n* = 3, *p* < 0.05. *, MDR2KO versus FVBN. # corticosterone versus vehicle. Scale bar, 100 μm.

IHC of C-type lectin domain family 4 member F (Clec4f), a marker of resident Kupffer cells, and C-C motif chemokine receptor 2 (Ccr2), a marker of recruited macrophages, were also measured in liver sections of male and female FVBN and MDR2KO mice treated with vehicle or corticosterone (Figure 7). Clec4f-immunolabeled cells were more abundant in MDR2KO than FVBN control mice of both genders (Figure 7A–C). Corticosterone treatment resulted in significant reduction of Kupffer cells in male, but not in female MDR2KO mice. In contrast, Ccr2-immunolabeled cells were increased in female MDR2KO mice but not in MDR2KO males compared to controls, and corticosterone treatment significantly reduced the accumulation of these cells in MDR2KO female mice livers (Figure 7D–F).

In summary, MDR2KO mice exhibited more than normal accumulation of monocytes/macrophages expressing CD68 or F4/80, with no difference between genders, and this immune response was reversed by corticosterone treatment. The amount of Clec4f^+^ Kupffer cells was also increased in both male and female MDR2KO mice and reversed by corticosterone treatment. However, Ccr2^+^ cells were increased only in female MDR2KO mice and corticosterone treatment did not reduce the amount of these cells. Because Ccr2 is the receptor for the CCL2 chemokine, these results suggest that in female MDR2KO mice, CCL2 may be secreted in response to MDR2KO-caused liver injury, inducing the migration and accumulation of monocytes/macrophages and other immune cells that express Ccr2 receptor on their surface.

### 2.6. Corticosterone Treatment Reduces Proinflammatory Cytokines in MDR2KO Mice

**Figure 8:**
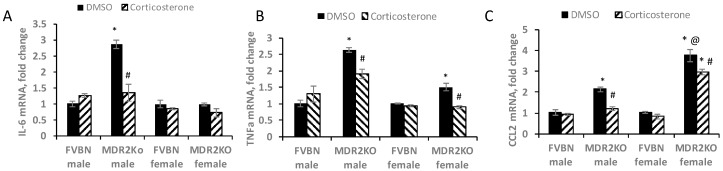
Proinflammatory cytokines are affected differently in females versus males MDR2KO mice when treated with corticosterone. The expression of several proinflammatory cytokines at RNA level was assessed by RT-qPCR, in RNA isolated from liver of males and females FVBN and MDR2KO mice which had been treated with vehicle or corticosterone. RT-qPCR was performed for IL-6 (**A**), TNFα (**B**) and CCL2 (**C**). *n* = 3–4, *p* < 0.05. * MDR2KO versus FVBN, # corticosterone versus DMSO, @ females versus males.

Several proinflammatory cytokines were assessed in livers of male and female MDR2KO mice as compared with FVBN control mice when treated with vehicle or corticosterone. As shown in Figure 8, RT-qPCR results indicated that interleukin 6 (IL-6) and tumor necrosis factor alpha (TNFα) were upregulated in male, but not female, MDR2KO mice as compared to FVBN controls and returned to normal expression levels by corticosterone treatment. C chemokine ligand 2 (CCL2) mRNA however was upregulated in female MDR2KO mice but not in males, and corticosterone treatment was less effective in reducing the expression of this proinflammatory chemokine.

### 2.7. Corticosterone Reverses Liver Fibrosis in MDR2KO Mice

**Figure 9:**
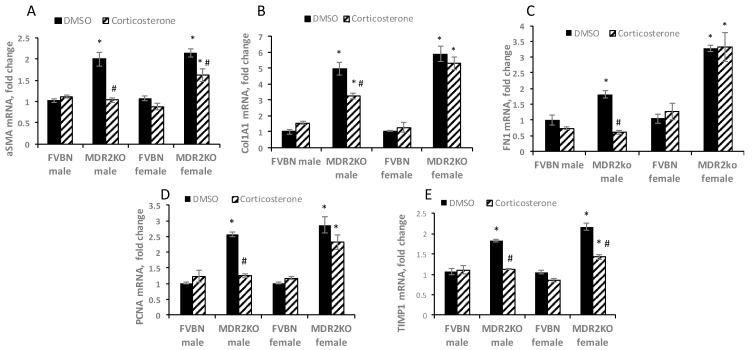
Corticosterone treatment reduces the expression of liver fibrosis markers to a greater degree in male compared to female MDR2KO mice. Total RNA was isolated from liver of male and female FVBN and MDR2KO mice treated with vehicle or corticosterone. RT-qPCR was performed for αSMA (**A**), Col1A1 (**B**), FN1(**C**), PCNA (**D**), and TIMP1 (**E**). *n* = 3–4, *p* < 0.05. * MDR2KO versus FVBN. # corticosterone versus DMSO.

The extent of liver fibrosis in male and female MDR2KO mice as compared to FVBN control mice when treated with vehicle or corticosterone, was further assessed. The expression levels of several hepatic fibrosis markers such as alpha smooth muscle actin (αSMA), collagen type 1 alpha 1 chain (Col1A1), fibronectin 1 (FN1), PCNA and tissue inhibitor of metalloprotease 1 (TIMP1) were tested by RT-qPCR (Figure 9). The results indicate that αSMA and TIMP1, which were two-fold upregulated in both male and female MDR2KO mice as compared to controls, were reduced to normal levels in males and only slightly decreased in females by corticosterone treatment. Col1A1, FN1 and PCNA mRNAs were upregulated in male and female MDR2KO mice, and corticosterone treatment resulted in decreased expression of these fibrosis markers in male, but not female, MDR2KO mice.

**Figure 10:**
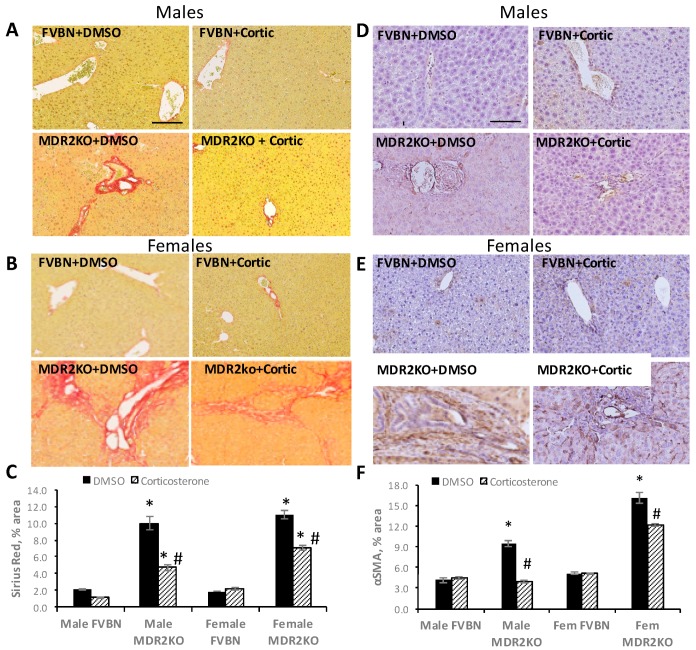
Corticosterone treatment reduces liver fibrosis in male and female MDR2KO mice. (**A**,**B**), Images of Sirius Red staining in liver sections from male (**A**) and female (**B**) FVBN and MDR2KO mice treated with DMSO or corticosterone. Plot of percent area of Sirius Red staining in livers from male and female FVBN and MDR2KO mice when treated with DMSO or corticosterone (**C**). αSMA IHC in the liver of male and female FVBN and MDR2KO mice in the absence and presence of corticosterone (**D**,**E**). Plot of αSMA IHC quantifications (**F**). * MDR2KO versus FVBN mice; # corticosterone versus DMSO treatment. @ females versus males; *p* < 0.05, *n* = 3–4. Scale bar, 100 μm.

We further assessed the degree of hepatic fibrosis in MDR2KO mice by Sirius Red staining of collagen in liver sections. As illustrated in Figure 10, panels A–B, Sirius Red staining was very extensive in the liver of all MDR2KO mice males and females as compared to FVBNs. Corticosterone treatment caused a strong reduction in collagen in the liver of male and female MDR2KO mice. The effect of corticosterone in reducing the hepatic deposits of collagen was greater in males than females, since Sirius Red staining was diminished by more than 50% in males, compared to 30% in females (Figure 10C). These results suggest that corticosterone is able to diminish the fibrosis process in liver of MDR2KO male and female mice, but it has a stronger effect in males than in females.

It is well-documented that a definitive marker of liver fibrosis is αSMA, which becomes increased in cholestasis due to activation of hepatic stellate cells (HSC) which largely contribute to producing increased levels of fibrillary collagen and αSMA during the fibrosis process [22,23]. In the present study, αSMA staining analyses demonstrated that αSMA protein was more abundant in the livers of MDR2KO mice than in controls (Figure 10D,E), in both males and females. Thus, there was 2.3-fold more hepatic αSMA in MDR2KO males than in FVBN control males, and a 60% reduction of αSMA staining in MDR2KO mice with corticosterone treatment. In MDR2KO females, corticosterone treatment reduced αSMA expression levels significantly, but to less of an extent than in MDR2KO males. These results suggest that there was an excess of αSMA protein in livers of MDR2KO mice as compared to FVBN mice, with females expressing more αSMA than males. Corticosterone treatment decreases αSMA accumulation in the liver of MDR2KO mice of both genders, but it has a greater effect in males than in females.

## 3. Discussion

In the present study we aimed to test the possibility of using glucocorticoids for the treatment of hepatic cholestasis in the MDR2KO mouse model of cholestasis. The results of this study demonstrate that the advanced biliary hyperplasia developed by MDR2KO mice of both genders by the age of two months is reversed by corticosterone treatment with high efficiency in males and only a moderate effect in females. We show that in addition to higher than normal cholangiocyte proliferation, the MDR2KO mice exhibited hepatic inflammation as indicated by a large accumulation of CD68^+^ and F4/80^+^ recruited monocytes/macrophages in livers of male and female MDR2KO mice. Interestingly, the increase of both types of cells, especially CD68^+^ cells, was more pronounced in females than males. The treatment with corticosterone lowered the number of these cells with no significant differences between males and females. Clec4f^+^ Kupffer cells were also equally increased in male and female MDR2KO mice as compared to controls, and this was reversed by corticosterone treatment. However, in liver of female, but not male, MDR2KO mice, the Ccr2^+^ cells were greatly increased as a result of significantly much higher secretion of CCL2 chemokine in female than male MDR2KO mice. Our data also demonstrate that MDR2KO mice exhibited liver fibrosis and this was more extensive in females than males. The assessment of corticosterone treatment on hepatic fibrosis showed that there was a significant reduction in collagen deposition and αSMA expression in the liver of MDR2KO mice of both genders. However, due to male MDR2KO mice displaying less fibrosis compared to female MDR2KO mice, corticosterone treatment allowed for male MDR2KO mice to have fibrosis measures closer to FVBN control levels compared to female MDR2KO mice. These data indicate that corticosterone treatment of hepatic cholestasis and fibrosis in MDR2KO mice resulted in a significant reversal of biliary hyperplasia, inflammation and fibrosis.

Many reports demonstrate that in MDR2KO mice, the ablation of the *AbcB4* gene, encoding the MDR2 protein, a hepatocyte membrane protein with function in phospholipid transport into the biliary ducts, results in hepatic cholestasis due to toxic bile accumulation [24,25]. This mouse model allows mechanistic studies at the cellular and molecular levels, providing data useful in finding treatments of human hepatic cholestasis caused by mutations in the *MDR3* gene, the human homolog for mouse MDR2. More than 30 mutations in *MDR3* have been reported [26], which are associated with biliary diseases such as intrahepatic cholestasis of pregnancy, low phospholipid-associated cholestasis and anicteric cholestasis [27,28,29]. In our study, MDR2KO mice exhibited cholangiocyte hyperplasia, increased hepatic inflammation and liver fibrosis compared to FVBN controls, which is in agreement with published data on these mice [16,17]. Our results also agreed with clinical observations in patients exhibiting low phospholipid-associated cholestasis, gallbladder cholesterol cholelithiasis, cirrhosis and other diseases caused by loss of function mutations of *MDR3*, the human homolog of the *AbcB4* mouse gene [27,28].

Analysis of changes in markers of biliary hyperplasia, hepatic inflammation and fibrogenesis in male and female MDR2KO mice suggested that there are strong differences between genders in regard to the extent of biliary cholestasis in this experimental model. Our data clearly demonstrate more advanced cholestasis and fibrosis in females as compared to males in MDR2KO mice, and this is consistent with previous reports which have also demonstrated gender-specific differences in hepatic injury in the MDR2KO mouse model [16,17,25,26]. Lammert et al., described spontaneous cholecysto- and hepatolithiasis in MDR2KO mice, with female mice displaying a higher gallstone-susceptibility [25]. Recently, Li et al. [30] described a role of the long noncoding RNA H19 in the mechanism of these gender-biased differences. Our data demonstrating that inflammatory hepatic cells, IBDM, Sirius Red-stained collagen and activated stellate cells were more pronounced in female than male MDR2KO mice are thus consistent with gender-related differences previously reported. Interestingly, a gender-biased susceptibility to hepatic cholestasis was also reported in clinical studies, the incidence of biliary dysfunctions in women being much higher than in men [31,32,33]. For example, primary biliary cirrhosis (PBC) is a cholestatic liver disease of autoimmune origin, which predominantly affects women (90% of patients) in middle age [31]. PBC is characterized by inflammatory destruction of the small intrahepatic bile ducts with fibrosis progressing to cirrhosis and subsequent liver failure. Studies on estrogen receptors (ER-α, β) demonstrated that ER-α and -β were observed in cholangiocytes of PBC patients but not in cholangiocytes from healthy controls [33].

Previous observations in human liver pathology and experimental animal models of liver diseases suggest that hepatic cholestasis is associated with a significant suppression of HPA axis activity [5,7,8,9,10,34]. The data presented here in the MDR2KO mouse model of hepatic cholestasis support the concept that the HPA axis is suppressed during cholestasis. Thus, the decreased level of serum corticosterone in MDR2KO mice is consistent with our previous observations in other animal models of hepatic cholestasis, i.e., bile duct ligation (BDL) and α-naphtylisothiocyanate (ANIT) feeding in the rat [9]. Reduced serum cortisol and corticosterone levels in BDL over a period of seven days and two weeks of ANIT-diet were also detected [9]. A low level of circulating CRH released from the hypothalamus found in MDR2KO mice is in agreement with significantly reduced serum CRH in rats after BDL procedure [9]. A clinical study on the expression of CRH in patients with intrahepatic cholestasis of pregnancy (ICP) after ursodeoxycholic acid (UDCA) treatment determined that CRH was significantly down-regulated in patients with ICP and that UDCA treatment resulted in an attenuation of serum CRH concentrations [34]. Our results from the MDR2KO model of hepatic cholestasis show lower than normal serum CRH in MDR2KO mice and these findings are in agreement with clinical data. To our knowledge, there are no reports on gender-dependent differences with regard to suppression of the HPA axis in patients with biliary cholestasis or other liver disorders so far. In our experimental MDR2KO mouse model, we observed a down-regulation of both corticosterone and CRH with the effect being more pronounced in male than female MDR2KO mice.

Only a few clinical studies looked at gender-biased responses to glucocorticoid (GC) treatment in hepatic cholestasis. A study on the effect of GCs on primary sclerosing cholangitis (PSC) indicated that a significant response to corticosteroid treatment was detected in 3.7% of all PSC patients in the study and this result was not gender-specific [35]. Another randomized double-blind study assessed the effect of two GCs, budesonide and prednisone, in combination with UDCA on PSC and only minor beneficial short-term effects of prednisone, but not budesonide, in addition to UDCA were found in PSC patients [36]. In an experimental model of hepatic cholestasis, we previously showed that glucocorticoid treatment significantly reduced biliary mass seven days after BDL surgery in rats [9]. Our results in the MDR2KO mouse model are consistent with the data obtained in the BDL rat model, demonstrating that corticosterone treatment results in strong reduction of cholangiocyte hyperplasia of MDR2KO male and female mice. As far as we know, this is the first study to demonstrate that corticosterone treatment causes a significant reduction of hepatic inflammatory cells and hepatic fibrosis in MDR2KO mice. Our study addresses the question of possible gender-dependent responses of cholestatic MDR2KO mice to corticosterone treatment, and the data suggest that in MDR2KO mice there is a gender-specific effect of corticosterone on indicators of cholangiocyte hyperplasia and liver fibrosis. Thus, the excessive bile duct mass in MDR2KO was significantly reduced after two weeks of corticosterone treatment in males and females, and corticosterone had a stronger effect in diminishing IBDM in males than in females. We demonstrate that in MDR2KO males, corticosterone treatment reduced IBDM by decreasing the cholangiocyte proliferation rate and not by inducing apoptosis. In the livers of MDR2KO females treated with corticosterone, the cholangiocyte proliferation remained unchanged and some apoptotic cells have been detected but the overall IBDM remained larger than in FVBN controls. The molecular mechanisms of these gender-biased effects of corticosterone on cholangiocyte proliferation in male and female MDR2KO mice, are still to be solved. Corticosterone treatment significantly reduced Sirius Red-stained collagen in all MDR2KO mice and it was more effective in males than in females. Corticosterone treatment reversed the abnormally increased expression of hepatic αSMA with a greater effect in males than in females. Even though not related to hepatic cholestasis, several reports described gender-related differences in the effect of GCs on expression of GR-regulated genes in animal studies [12,13,37] as well as clinical investigations [37,38]. Our data indicated that this gender-specific response to corticosterone was not due to differences in GR expression or intracellular distribution in MDR2KO mice as compared to FVBN controls, but may be due to a disparity in the downstream effectors or GR activation, or may be a reflection of a disparity in the severity of cholestatic symptoms in male versus female MDR2KO mice. Further studies to determine the cause of the gender difference in response to glucocorticoid treatment are warranted.

In regard to the anti-inflammatory effect of low-dose corticosterone treatment in the MDR2KO mouse model of hepatic cholestasis, our data show that corticosterone has a positive and effective outcome, mediating a major reversal of macrophage accumulation within periportal and periductal areas of the liver in MDR2KO mice. We measured the accumulation of several types of immune cells such as CD68^+^, F4/80^+^, Clec4f^+^ and Ccr2^+^ in liver of MDR2KO mice as compared to FVBN controls. CD68 is a marker for recruited monocytes/macrophages which take part in the inflammatory response to liver injury by releasing cytokines such as TNFα, IL-1 and IL-6 [19,21,39]. The F4/80 marker is also expressed in a large group of macrophages, which may or may not express CD68 [20]. In our study, corticosterone was able to strongly diminish the abnormally high number of CD68^+^ and F4/80^+^ macrophages in liver of MDR2KO mice, regardless of gender. We also assessed Kupffer cells, which are hepatic resident mononuclear cells (MNC) with very heterogeneous lineages [20,40]. Because Clec4f, a C-lectin protein, is specifically located in the plasma membrane of Kupffer cells [41], we assessed the amount of Clec4f^+^ cells in the liver of MDR2KO mice versus FVBN controls. Clec4f^+^ macrophages were increased in both male and female MDR2KO mice, and this abnormal excess was reversed by corticosterone treatment. Ccr2 is known to be expressed on the surface of many types of immune cells such as monocytes and T-cells which are chemo-attracted by the specific ligand CCL2 [42]. Unlike all the other immune cells tested in our study, Ccr2^+^ cells were greatly increased in female but not male MDR2KO mice as compared to controls, and corticosterone treatment did not reduce this immune response. It is well-known that immune cells which exhibit Ccr2, the receptor for CCL2 chemokine, migrate along the CCL2 gradient to the damaged cells in the liver [42]. Interestingly, when CCL2 expression was measured in livers of male and female MDR2KO mice and controls, there was two-fold more CCL2 mRNA in female than in male MDR2KO mice. Many Ccr2^+^ macrophages were detected in close proximity to cholangiocytes, lining the ductal epithelium in female but not male MDR2KO mice. These findings point to an important role of Ccr2^+^ recruited cells, in the pathology of liver in MDR2KO female mice.

The use of corticosteroids for liver disease treatment has been long studied [43]. Clinical case studies on GC treatment of chronic cholestasis, such as autoimmune cholangitis, drug-induced cholangitis and intrahepatic cholestasis of pregnancy have been reported [44]. It has been demonstrated that in cases of prolonged cholestatic jaundice after endoscopic retrograde cholangiography, prednisone treatment resulted in progressive restoration of serum bilirubin and alkaline phosphatase to normal levels [44,45]. Interestingly, many case studies focused on prolonged cholangitis following removal of gallstones in women specifically, since estrogens are thought to promote gallstone formation [45]. Observational studies show that estrogen therapy is an important risk factor in gallbladder disease [46,47]. Studies on experimental models also suggested a beneficial effect of GC treatment, such as dexamethasone, for attenuation of ductular impairment [48,49]. Our data from the MDR2KO mouse model, showing cholangiocyte hyperplasia and hepatic fibrosis being more pronounced in females than in males, and being partially reduced by corticosterone treatment, are in agreement with these reports.

GCs are known to have anti-inflammatory effects in many types of immune conditions by inducing apoptosis of inflammatory cells and suppressing proinflammatory genes encoding cytokines, chemokines and cell adhesion molecules, thereby reducing the inflammation process [50]. In an experimental model of contact dermatitis for example, dexamethasone reduced the number of CD68^+^ macrophages [51]. The same effect was found when allergic airway inflammation was induced in mice, with CD68^+^ macrophages in the lungs being significantly diminished with dexamethasone treatment [52]. Interestingly, GC treatment was reported to remarkably reduce the macrophage content of atherosclerotic plaques in an experimental model of atherosclerosis in which macrophages are crucial for inducing blood vessel inflammation [53]. Thus, our data in MDR2KO mice, where CD68^+^, F4/80^+^ and Clec4f^+^ macrophages in cholestatic liver were reduced by corticosterone treatment, are in line with reported results in which GCs were shown to reduce the accumulation of macrophages associated with inflammation in various parts of the body.

## 4. Materials and Methods

### 4.1. Materials

All chemical reagents were purchased from Sigma-Aldrich (St. Louis, MO, USA) and were of the highest grade available, unless otherwise specified. The antibodies used in IHC experiments were: CK19, CD68, F4/80, αSMA, Clec4f, Ccr2, PCNA from Abcam (Cambridge, MA, USA); GR antibody was from Santa Cruz Biotechnology (Dallas, TX, USA).

### 4.2. Animal Treatment

FVBN and MDR2KO mice were purchased from Charles River and maintained in a temperature-controlled environment at 20–22 °C with a 12:12 h light-dark cycle, having free access to food and drinking water. All animal procedures were performed in accordance with the approval of the Baylor Scott & White Health Institutional Animal Care and Use Committee, (protocol no. 2015-015, approved 8 December 2015). Two-month-old male and female FVBN and MDR2KO mice were treated with corticosterone (1 mg/kg/day via ip implanted osmotic minipumps Alzet (Cupertino, CA, USA) or Vehicle (20% DMSO) for 2 weeks, following manufacturer’s instructions. Each treatment group consisted of 4–5 animals.

### 4.3. Assay of HPA Axis Activity 

The concentration of corticosterone and CRH in the serum of male and female FVBN and MDR2KO mice were measured by using EIA kits from Phoenix Pharmaceuticals (Phoenix, AZ, USA), according to manufacturer’s instructions.

### 4.4. Assessment of Cholangiocyte Proliferation, Liver Fibrosis and Inflammation

In order to measure the amount of IBDM in wildtype and MDR2KO mice in the absence or presence of corticosterone, IHC on paraffin-embedded liver sections was performed using a CK19 antibody for labeling the cholangiocytes, followed by VectaStain ABC kit from Vector Laboratories Inc. (Burlingame, CA, USA) for color development, following previous described protocols [9]. Liver sections of 4 μm were obtained with a Leica microtome. The VectaStain-stained sections were mounted on slides, and scanned with a Leica SCN400 scanner, with 20× optical magnification. Screenshots with 10× digital magnification were taken and used for quantification of field-stained pixel percentage of CK19-positive cells. The same procedure was employed for IHC of CD68, F4/80, Clec4f, Ccr2 and αSMA in liver sections. The images were analyzed with ImageJ software downloaded from the NIH website. The degree of fibrosis in FVBN and MDR2KO mice treated with vehicle or corticosterone was assessed by staining collagen I and III fibers in liver sections with Sirius Red (kit from Sigma Aldrich, St. Louis, MO, USA) and then processing the slides in the same manner used for IHC analysis. Apoptosis in the liver of FVBN and MDR2KO mice was assessed using a TUNEL kit from Abcam (Cambridge, MA, USA) according to manufacturer’s instructions and was conterstained with methyl green.

### 4.5. Assays of mRNA Expression of Genes with Role in Inflammation and Fibrosis by RT-qPCR

Changes in mRNA expression of TNFα. IL-6, CCL2, αSMA, COL1A1, FN1, TIMP1 and PCNA were estimated by performing RT-qPCR. Total RNA was isolated from liver tissue of FVBN and MDR2KO mice treated with vehicle or corticosterone, by using RNeasy kit from Qiagen (Germantown, MD, USA) according to manufacturer’s instructions. The reverse transcription of RNAs was done with iScript RT Supermix from Bio-Rad (Hercules, CA, USA), and then the real time PCR assay was performed with iTaq Universal SYBR Green Supermix from Bio-Rad, and corresponding primers from SABiosciences-Qiagen. The amplification reactions were run with an AriaMx Real Time PCR system from Agilent Technologies (Santa Clara, CA, USA).

### 4.6. Statistical Analysis of Results

Quantifications obtained in all experiments were subjected to statistical analysis, i.e., the average and standard error of the mean (SEM) were calculated for each group of animals; namely male FVBN treated with vehicle (DMSO), females FVBN treated vehicle, FVBN males with corticosterone, FVBN females with corticosterone, MDR2KO males treated with vehicle, MDR2KO females treated with vehicle, MDR2KO males with corticosterone, and MDR2KO females treated with corticosterone. When comparing two groups, the significance was calculated using Student’s *t*-test. We also used two-way ANOVA followed by an appropriate post-hoc test on GraphPad Prism (San Diego, CA, USA) when comparing multiple groups. The statistical difference was considered significant when the *p* value was less than 0.05.

## 5. Conclusions

In conclusion, we demonstrated, in a hepatic cholestasis mouse model, that treatment with corticosterone to activate the HPA axis reversed cholangiocyte proliferation, inflammation and fibrosis of the liver in MDR2KO mice, having a higher efficacy in males than in females. Overall, the corticosterone treatment was beneficial in reducing liver inflammation and fibrosis in this mouse model of hepatic cholestasis in both males and females. Future studies on combining corticosterone treatment with other factors, novel or known to repress the damaging effects of biliary cholestasis, may improve the healthcare procedures for treating biliary diseases.

## Abbreviations

ANIT	α-naphtylisothiocyanate
αSMA	Alpha smooth muscle actin
BA	Bile acid
BDL	Bile duct ligation
CCL2	C chemokine ligand 2
Ccr2	C chemokine receptor 2 (receptor of CCL2)
Clec4f	C-lectin 4f
CK19	Cytokeratin 19
Col1A1	Collagen type 1 alpha 1
CRH	Corticotrope releasing hormone
EIA	Enzyme immune assay
ER-α, ER-β	Estrogen receptor-α, -β
FN1	Fibronectin 1
GR	Glucocorticoid receptor
HPA	Hypothalamus-Pituitary-Adrenal axis
HSC	Hepatic stellate cells
HSP	Heat shock protein
IBDM	Intrahepatic biliary duct mass
IHC	Immunohistochemistry
IL-6	Interleukin 6
MDR2KO	Multidrug resistance protein 2 knockout transgenic mouse
MNC	Mononuclear cells
PCNA	Proliferating cell nuclear antigen
PSC	Primary sclerosing cholangitis
PBC	Primary biliary cirrhosis
TIMP1	Tissue inhibitor of metallopeptidases 1
TNFα	Tumor necrosis factor alpha
UDCA	Ursodeoxycholic acid
